# Supplementary material for: An International Consensus on the Design of Prospective Clinical–Translational Trials in Spatially Fractionated Radiation Therapy for Advanced Gynecologic Cancer
Source: Cancers (Basel). 2022 Aug 31;14(17):4267. doi: 10.3390/cancers14174267 (PMC9454841; doi:10.3390/cancers14174267)
Supplement: Supplementary file 1 [file cancers-14-04267-s001.zip › cancers-14-04267-s001/Amendola_File S1_ClinTrial Cons Gyn_Literature Summary (FNL).pdf]

File S1

Literature Summary Table

SFRT for Cervical Cancer

This collated literature table presents, for your reference, a summary of major pertinent studies that were considered in developing these recommendations. This summary table summarizes both studies of multiple tumor sites that include cervical cancer patients, one other gynecologic tumor site and disease-specific series of only cervical cancer patients. The table is structured based on study type and objective, patient selection, SFRT and conventional radiation therapy parameters, and treatment outcome criteria.

**Abbreviations:**	
gr	= grade
LC	= local control
LR	= local recurrence
DSS	= disease-specific survival
PFS	= progression-free survival
OS	= overall survival
Tox	= toxicity
yr, yrs	= year, years
pt, ptss	= patient, patients
LRT	= Lattice therapy
GRID	= GRID therapy

**Abbreviations:**	
RR	= response rate
PR	= partial response
CR	= complete response
NR	= no response
cCR	= clinical complete response
pCR	= pathologic complete response
cERT	= Conventional radiation
fr	= fraction
n/a	= not applicable
–	= no data
*	= per author’s communication

Cervical Cancer Specific Studies						
**Auth, Year**	**Pt No. Sites**	**Objectives**	**Methods**	**Results**	**Dose/ Spatial Fx**	**Conclusion**
Amendola 2010 (Cureus 2(9): e15)<br><br>Treated: 2009*	**1**	**Cervix ca**	Study type: Case report<br><br>Study Population: Bulky adv cervix ca, Stage IIB, pelvic node +, Severe bleding Tu vol: 250 cm^3<br><br>Outcome Measures: RR, LC, toxicity<br><br>Technique: LRT (VMAT)<br><br>Follow-up: 11 months*	RR: 70% tumor reduction (250 cm^3 to 75 cm^3) (at end of cERT Pathologic CR (hysterectomy 6 week after cERT<br><br>LC: local control at 11 mo.*<br><br>DSS/OS: Death of distant (lung metastases) 11 mo. post-therapy *<br><br>Toxicity: grade 2 diarrhea No late toxicity	LRT sequencing: sequential, concomitant: First 6 Gy/2 fr cERT (for severe bleeding). Then concomitant LRT (to gross tu volume) and cERT to pelvis.<br><br>LRT method: Vertex size: 1 cm Vertex number: 15 Vert distance: 3 cm (average)<br><br>LRT dose: Vertex dose: 48 /20 Periph dose: 36/20 (periphery/pelvis)<br><br>cERT dose: 6/2 and 36/20<br><br>OAR dose: Configured to avoid OARs<br><br>Concurr tx: wkly Cisplatin x 6	Rapid response. No residual tumor at TAH/BSO (pCR).<br><br>Relatively low and fractionated dose in this first case of LRT in cervical cancer.<br><br>Low toxicity.

Auth, Year	Pt No. Sites	Objectives	Methods	Results	Dose/ Spatial Fx	Conclusion
Amendola 2020<br><br>(Radiation Research, Epub 2020 https://meridian.allenpress.com/radiation-research/publish-ahead-of-print)<br><br>Treated:<br>2013-2019*	**10**	**Cervix ca**<br><br>Definitive treatment	Study type:<br>Retrospective<br><br>Study Population:<br>Bulky advanced cervix ca, stage IIIB, IVA<br>7 squamous cell ca,<br>3 adeno ca<br><br>Outcome Measures:<br>RR, LC,<br>imaging response,<br>DSS/OS,<br>toxicity<br><br>Technique:<br>LRT (VMAT)<br><br>Follow-up<br>median 16 (1-77) mo.	RR:<br>Mean tumor regression by 51% at end of cERT<br><br>Metabolic response (PET/CT) 89% (9/10 pts).<br><br>CR 78%, PR 22%<br><br>LC: 100%<br><br>DSS: 53%<br><br>OS: 47%*<br><br>Toxicity:<br>No gr >2 early or late toxicity	LRT sequencing:<br>LRT first<br><br>LRT method:<br>Vertex size: 1.05 cm (average)*<br>Vertex number: 5 (average)<br><br>LRT dose:<br>Vertex dose: 24/3 (GTV)<br>Periph dose: 9/3<br><br>cERT dose:<br>mean 44.28 (39.6 - 45.0) /22-25<br><br>OAR dose:<br>per RTOG criteria*<br><br>Concurr tx:<br>wkly Cisplatin x 6; including LRT fraction*	High metabolic response and high local control rate.<br><br>Rapid and variable intra-treatment CT-based response.<br><br>Low toxicity despite concurrent chemotherapy that included the SFRT fraction.

Other Gynecologic Tumor Site

Auth, Year	Pt No. Sites	Objectives	Methods	Results	Dose/ Spatial Fx	Conclusion
Blanco Suarez 2015<br><br>(Cureus 7(11): e389)<br><br>Treated:<br>2013	1	**Ovarian ca** (MMT)<br><br>Local control for post-surgical / chemotherapy recurrence of initial stage IIIC ovarian Mixed Mullerian tumor	Study type:<br>Case report<br><br>Study Population:<br>Bulky 14 cm, 1496 cm^3 pelvic/abdominal recurrence from Mixed Mullerian tumor of the ovary with psoas muscle invasion<br><br>Outcome Measures:<br>RR, LC, DSS, OS, Tox<br><br>Technique:<br>LRT (VMAT)<br><br>Follow-up:<br>21 months	RR: Tumor volume decrease by 70%<br><br>LC:<br>1/1 – size decrease by 70%<br><br>DSS: 1/1 – stable disease in all treated lesions for 19 months<br><br>Death of metastatic progression at 21 months post-therapy; local control<br><br>OS: Death of distant metastases<br><br>Toxicity:<br>Minimal	LRT sequencing:<br>LRT first<br><br>LRT method:<br>Vertex size: 1.5-2 cm<br>Vertex number: 12<br><br>LRT dose:<br>Vertex dose: 27/3<br>Periph dose: 9/3<br><br>cERT dose:<br>1.8 Gy x 5 fr<br>2 Gy (peripheral dose) + 5 Gy as SIB x 5 fr<br>1.8 Gy x 5 fr<br>2 Gy (peripheral dose) + 5 Gy (strips) x 5 fr<br><br>OAR dose:<br>Configured to avoid OARs<br><br>Concurr tx:<br>None.<br>Extensive prior chemotherapy	Excellent response in a high-volume recurrent therapy-refractory tumor and prolonged local control. Minimal toxicity.

Multiple-site Studies including Gynecologic Cancer (not otherwise specified as cervical cancer) Patients

Author, Year	Pt No. Sites	Objectives	Methods	Results	Dose/ Spatial Fx	Conclusion
Mohiuddin M et *al.*<br>(Radiat Oncol Invst 1996; 4:41-7)<br><br>Treated:<br>~1990-1995	61<br>(72 sites)<br><br>GI: 18<br>Sarcoma: 12<br>GU: 9<br>**Gyn: 9**<br>Melanoma: 5<br>H&N (SCCa): 4<br>Lung: 1<br>Breast: 2<br>Thyroid: 4	Multiple sites<br><br>Palliative only	Study type:<br>Clinical trial<br><br>Study Population:<br>Palliative only tx refractory<br>Primarily large soft tissue masses. 44/72 pts abdomen/pelvis<br>24% (17 sites) had prior RT (12.6-79 Gy)<br><br>Outcome Measures:<br>Palliation (pain, bleeding, mass effects): RR, CR, PR, NR<br>Tox (EORTC grading)<br><br>Technique:<br>GRID therapy: Block<br><br>Follow-up:<br>0.5-28 mo (d/t adv stg)<br>10 pts alive $\geq$ 1 yr	RR: 91%<br><br>LC: Durable response in most pts w longer survival.<br>GRID $\geq$15 Gy: 100% vs 79% RR<br>cERT $\geq$40 Gy: 100% vs 92% RR<br><br>DSS: –<br><br>OS:<br>27/71 pts: 3-28 mo.<br>10/71 pts: survived >1 yr<br><br>Toxicity:<br>No grade 2 or higher tox<br>No bowel tox despite 44 pts w tx to abdomen/pelvis<br>(1 bowel obstruction due to tumor at laparotomy)	GRID sequencing:<br>GRID only: 44%<br>GRID generally first (40/72, pts with life expectancy of >1 mo.): GRID + cERT<br><br>GRID method:<br>Block (50% open)<br>6, 24MV<br>Single field<br><br>GRID dose: 10-15/1 (for GRID+ cERT)<br>15-25/1 for GRID only) to Dmax<br><br>cERT dose: (in 44/72) wide range; 78 Gy<br><br>Dose to periphery: –<br><br>OAR dose: –<br><br>Concurr tx: No	GRID therapy results in high (>90%) symptomatic tumor response rate, with minimal toxicity.<br><br>Dose response relationship: High cumulative GRID and cERT doses are needed for satisfactory CR rates:<br>GRID dose $\geq$15 Gy: higher RR, CR,<br>cERT DRR $\geq$40 Gy: higher RR, CR.<br><br>Response by tumor type: Best RR in sarcoma (94%) and SCCa (92%); least RR in adenocarcinoma (69%).<br><br>Parallelism of GRID therapy with brachytherapy, enabling delivery of high doses to small volumes with modest doses over a larger volumes of tissue.

Author, Year	Pt No. Sites	Objectives	Methods	Results	Dose/ Spatial Fx	Conclusion
Mohiuddin M et *al.*<br>(IJROBP 1999;45:721-7)<br><br>Treated: 1/1995-3/1998	71<br>(87 sites)<br><br>Overall sites:<br>Lung: 18<br>H&N: 17<br>Sarc: 10<br>GI: 4<br>GU: 5<br>**Gyn: 8**<br>Skin: 11<br>Melan: 3<br>Breast: 3<br>Thyr: 2<br>UNK:4<br>Liver: 2	Multiple, Palliative 89%<br><br>Curative: +/- surgery 11%	Study type:<br>Retrospective<br><br>Study Population:<br>Palliative: 89% (63/71)<br>Advanced, definitive:<br>H&N, 11% (8/71)<br><br>Tumor >8 cm<br><br>Prior RT: 9% (8/87 sites)<br><br>Outcome Measures:<br>RR<br>Pts who died during/within 1 mo. of tx (7) inevaluable for RR, but included in toxicity analysis.<br>Path response (8 H&N ca. pts)<br><br>Technique:<br>GRID therapy: Block<br><br>Follow-up:<br>7 (3-42) mo.	RR: 76%<br>Palliative pts: 78%<br><br>RR overall:<br>GRID dose >15 Gy:<br>RR 94 vs 62% (p-.002)<br><br>cERT DRR >40 Gy:<br>0 Gy: 86%, 0% (RR, CR)<br><40 Gy: 91%, 13% (RR, CR)<br>≥ 0 Gy: 94%, 24% (RR, CR)<br><br>Estimate of RR in gyn tumors:<br>Pelvic rumors:<br>RR 50%, CR ~12%<br><br>LC: –<br><br>DSS: –<br><br>OS: –<br><br>Toxicity (overall):<br>No short or long-term GI toxicity	GRID sequencing:<br>Only: 20% (14/71)<br>GRID, then cERT 66%(47/91)<br><br>GRID method:<br>Block (50% open)<br>6, 18 MV<br><br>GRID dose: 10-20 Gy/1 median: 15 Gy/1 to 10-12 Gy (for prior RT), at Dmax<br><br>cERT dose:<br>Definitive pts (8 H&N):<br>50-70 Gy<br>Palliative pts: –<br><br>Dose to periphery: –<br><br>OAR dose: –<br><br>Concurr tx: No	High response, low toxicity.<br><br>Dose response relationship: Validating the results from Mohiuddin et *al.* (Radiat Oncol Invst 1996):<br>GRID dose >15 Gy:<br>Higher RR.<br><br>cERT DRR >40 Gy:<br>Higher RR, CR.<br><br>Response by tumor site:<br>SCCa had better CR (29%).<br>Sarcoma (11%) had lower RR, larger tumors (>20 cm) and early death.

Author, Year	Pt No. Sites	Objectives	Methods	Results	Dose/ Spatial Fx	Conclusion
Neuner G<br>et *al.*<br>(IJROBP<br>2012;82(5):1642-9)<br><br>Treated:<br>2003-2008	79<br><br>Lung: 18<br>H&N: 14<br>Sarcoma:14<br>Liver: 6<br>Skin: 5<br>Breast: 4<br>Colon/<br>Anus: 5<br>Kidney: 3<br>Thyroid: 3<br>Esoph: 2<br>Lymph: 2<br>Prostate:1<br>**Ovary: 1**<br>Unknown:1	Multiple, Palliative 77%<br>Most lung, H&N<br><br>Curative: 23%<br>Most lung,H&N<br>Pre-op RT<br>4 pts	Study type:<br>Retrospective review<br><br>Study Population:<br>Bulky, median 7.6 cm (4-10 cm)<br>Most lung, H&N, Sarc<br><br>Most common tx site: neck<br><br>Outcome Measures:<br>Symptom response:<br>CR= complete resolution<br>PR= any improvement<br>NR= no improvement or progression<br>Imaging response (n=40): RECIST<br><br>Technique:<br>GRID therapy: Retrospective comparison of Block vs. MLC<br><br>Follow-up:<br>2 (0-51.6) mo.<br>28% (22 pts) lost to f/u	RR: Block vs MLC<br>Pain: 75% 74%<br>Mass eff: 67% 73%<br>Bleeding: 50%, 80%<br>Other symptoms: high response.<br><br>Imaging RR (CR+PR):<br>Block vs MLC<br>27% 32%<br>LC: –<br><br>DSS: –<br><br>OS: 29% (23/79)<br>(study not intended to report survival)<br><br>Median survival:<br>2.2 mo. (Block)<br>4.1 mo. (MLC), p=NS<br><br>Toxicity:<br>2 early gr4 (skin)<br>3 late gr3-4 (skin)<br><br>*Early:*<br>2 pts gr 4 dermatitis<br>*Late:*<br>overall: 3 pts: late gr3-4: chronic skin ulcer (2/3 pts with skin involvement)<br>No GI tox reported.	GRID sequencing:<br>Only: 20%(palliative pts)<br>First: 72%, with 1-2 day gap to cERT<br>In early cERT: 8%<br><br>GRID method:<br>- Block<br>- MLC: average open/ closed ratio 0.31.<br><br>GRID dose: 10-20 Gy (median 15 Gy)/ 1 fr<br>Block: at Dmax<br>MLC: GTV, no expansion<br><br>cERT dose: >35-40 Gy,<br>No dose reduction for GRID, (e.g. 70.2 Gy for H&N), but normal tissue dose reduction<br><br>Dose to periphery: –<br><br>OAR dose: Blocking of neural structures, kidney, GI tract, heart; minimizing exit dose<br><br>Concurr tx:<br>None (only in H&N ca/curative)	High symptom response rate; no difference in response between Block vs MLC based GRID.<br><br>No difference in imaging response for Block vs MLC based GRID therapy.<br><br>Low toxicity rates.<br><br>Ease and efficacy of MLC-based GRID may enable more widespread adoption of SFRT.
