# Supplementary material for: An International Consensus on the Design of Prospective Clinical–Translational Trials in Spatially Fractionated Radiation Therapy for Advanced Gynecologic Cancer
Source: Cancers (Basel). 2022 Aug 31;14(17):4267. doi: 10.3390/cancers14174267 (PMC9454841; doi:10.3390/cancers14174267)

File S2

Aggregated Voting Results: SFRT Clinical Trial Consensus for Cervical Cancer

Rating scale and Voting categories: 1,2,3=not appropriate -- 4,5,6=may be appropriate -- 7,8,9=appropriate

Column **Vote category** denotes vote category (*Appr* = Appropriate, *May be appr* = May be appropriate, *Not appr* = Not appropriate) that received the highest percentage of votes. The percentage agreement with the vote category is shown in column **%Agreement with Vote**.

Column **Agreement** denotes level of agreement/consensus based on the voting: *high*, *moderate* or *low* as defined in the table below:

Agreement	Definition
High	Percent agreement $\geq$ 67% AND if any disagreement, it is by at most 1 voting category
Moderate	60-67% agreement OR agreement $\geq$67% but votes in both *Appropriate* and *Not appropriate* voting categories
Low	Percent agreement <60%

Column **Expert Panel Consensus**: denotes resulting consensus (*high*, *moderate* or *low*) based on the Expert Panel's review of the voting rounds and their deliberations.

Column **Expert Panel Conclusions** denotes additional conclusions by the Expert Panel not contained in the criteria and consensus results.

Abbreviations:
- Exp. Panel = Expert Panel
- Nr = number
- OAR = organ at risk
- RT = radiation therapy

	Vote								Expert Panel	
**Clinical Trial Criteria**	**Nr of Votes**	**Min Rank**	**Max Rank**	**Range of ranks**	**Mean Rank**	**%Agreement with Vote**	**Vote Category**	**Agreement**	**Exp. Panel Consensus**	**Exp. Panel Conclusions**

1	**Eligible Stage, Tumor size: Patients with these tumors should be allowed to enroll in an SFRT trial**										
	FIGO Stage IB2-IIB, >7 cm	4	4	9	5	6.8	50%	Appr<br>May be appr	low	moderate	Agreed. In addition, tumors ≥6 cm are eligible. Patients with tumors ≥5 cm who are unsuitable for brachytherapy (e.g. severe anatomic distortion such as obstructing uterine fibroid or other uterine abnormalities; anatomical or tumor-related severe vaginal atrophy/stenosis; ASA grade IV medical comorbidities) can be considered.
	Comment: Consider including tumor size ≥5 cm	1									
	FIGO Stage III-IVA, >7 cm	4	7	9	2	7.8	100%	Appr	high		
	Comment: Consider including tumor size ≥5 cm	1									

	Vote								Expert Panel	
**Clinical Trial Criteria**	**Nr of Votes**	**Min Rank**	**Max Rank**	**Range of ranks**	**Mean Rank**	**%Agreement with Vote**	**Vote Category**	**Agreement**	**Exp. Panel Consensus**	**Exp. Panel Conclusions**

1	**Eligible Stage, Tumor size: Patients with these tumors should be allowed to enroll in an SFRT trial** – continued										
	Pelvic node-positive or node-negative	4	3	9	6	5.8	50%	Not appr Appr	low	high	Not agreed/reconciled. Patients with both involved and uninvolved pelvic lymph nodes are eligible.
	Comment: For large nodes >5 cm LRT feasible. Consider simultaneous integrated boost (SIB) during cERT.	1									
	Para-aortic node-positive or node-negative	4	2	9	7	5.5	50%	Not appr Appr	low	high	Not agreed/reconciled. Patients with both involved and uninvolved para-aortic lymph nodes are eligible.
	Comment: For large nodes >5 cm LRT feasible. Consider SIB during para-aortic cERT.	1									
	Supraclavicular node-positive (no other metastases)	4	3	9	6	5.0	50%	May be appr	low	high	Not agreed. Patients with involved supraclavicular lymph nodes are excluded because supraclavicular node involvement generally signifies distant metastases and because such patients are generally excluded from clinical trials of *potentially curable* advanced cervical cancer.
	Comments: 1. This would give an opportunity to evaluate abscopal effect 2. As palliative treatment in the primary tumor.	2									

	Vote								Expert Panel	
**Clinical Trial Criteria**	**Nr of Votes**	**Min Rank**	**Max Rank**	**Range of ranks**	**Mean Rank**	**%Agreement with Vote**	**Vote Category**	**Agreement**	**Exp. Panel Consensus**	**Exp. Panel Conclusions**

1	**Eligible Stage, Tumor size: Patients with these tumors should be allowed to enroll in an SFRT trial** – continued										
	Other Eligible Stage, Tumor size	2							N/A	<u>Not agreed</u>. Metastatic disease should be excluded from a trial of primary locally advanced cervical cancer.
	Comments:<br>1. Consider other metastatic sites (not visceral).<br>2. Palliative									

Clinical Trial Criteria	Vote: Nr of Votes	Min Rank	Max Rank	Range of ranks	Mean Rank	%Agreement with Vote	Vote Category	Agreement	Expert Panel: Exp. Panel Consensus	Exp. Panel Conclusions

2	Eligible Histology: Patients with these histologies should be allowed to enroll in an SFRT trial										
	Squamous cell carcinoma	4	7	9	2	8.0	100%	Appr	high	high	Agreed.
	Adenocarcinoma	4	4	9	5	6.8	75%	Appr	high	high	Agreed.
	Comment: In our experience adeno and adenosquamos carcinomas treated with LRT had good local control but not overall survival.	1									
	Adenosquamous carcinoma	4	4	9	5	6.8	75%	Appr	high	high	Agreed.
	Comment: Same as above.	1									
	Both HPV-positive and HPV-negative tumors	4	7	9	2	8.0	100%	Appr	high	high	Agreed.

	Vote								Expert Panel	
**Clinical Trial Criteria**	**Nr of Votes**	**Min Rank**	**Max Rank**	**Range of ranks**	**Mean Rank**	**%Agreement with Vote**	**Vote Category**	**Agreement**	**Exp. Panel Consensus**	**Exp. Panel Conclusions**

3	**Eligible Age**										
	>18 years old	4	5	9	4	7.0	75%	Appr	high	high	Agreed.
	<85 years old	4	1	8	7	5.2	50%	Appr	low	high	Agreed.
	Comment: Depends on patient's general status.	1									
	No upper age limit	4	7	9	2	7.8	100%	Appr	high	high	Agreed.
	Comments: Depends on patient's general status.	2									Patients 85 years and older with good performance status are also eligible.

	Vote								Expert Panel	
**Clinical Trial Criteria**	**Nr of Votes**	**Min Rank**	**Max Rank**	**Range of ranks**	**Mean Rank**	**%Agreement with Vote**	**Vote Category**	**Agreement**	**Exp. Panel Consensus**	**Exp. Panel Conclusions**

4	**Stratifications**										
	Lymphnodes: positive vs. negative	4	4	9	5	6.2	50%	Appr<br>May be appr	low	high	Agreed.
	Squamous cell carcinoma vs. Adenocarcinoma	4	5	9	4	7.2	75%	Appr	high	moderate	Not agreed. Despite potential differences in outcome, squamous cell carcinoma vs. adenocarcinoma is generally not stratified in clinical trials of locally advanced cervical cancer.

	Clinical Trial Criteria	Vote: Nr of Votes	Vote: Min Rank	Vote: Max Rank	Vote: Range of ranks	Vote: Mean Rank	Vote: %Agreement with Vote	Vote: Vote Category	Vote: Agreement	Expert Panel: Exp. Panel Consensus	Expert Panel: Exp. Panel Conclusions
5	**EXCLUSION Criteria: Patients with these histologies sites should be EXCLUDED from an SFRT trial**										
	Small cell neuroendocrine ca.	4	1	8	7	4.8	50%	Appr<br>Not appr	low	high	Patients with unusual or radiosensitive histologies, such as sarcoma, neuroendocrine small cell carcinoma or lymphoma, are not eligible.
	Comments:<br>1. Depending on the size, if voluminous clinically treatable with SFRT.<br>2. Rare or infrequent histologies must be excluded.<br>3. Very chemo-sensitive.	3									
	Lymphoma	4	1	9	8	6.2	75%	Appr	moderate	high	
	Comments:<br>1. Exclude in-frequent histologies.<br>2. Very chemo-sensitive.	2									
	Sarcoma	4	1	3	2	1.5	100%	Not appr	high	high	
	Comments:<br>1. Carcinosarcoma would benefit from lattice RT<br>2. Exclude in-infrequent histologies	2									

	Vote								Expert Panel	
**Clinical Trial Criteria**	**Nr of Votes**	**Min Rank**	**Max Rank**	**Range of ranks**	**Mean Rank**	**%Agreement with Vote**	**Vote Category**	**Agreement**	**Exp. Panel Consensus**	**Exp. Panel Conclusions**

6	**EXCLUSION Criteria: These conditions should be EXCLUDED from an SFRT trial**										
	Recurrent tumors (after prior radiation)	4	2	8	6	4.5	50%	May be appr	low	high	Not agreed/reconciled**.** Patients with recurrent tumors after prior radiation are not eligible.
	Comments:<br>1. Clinically treatable with SBRT/LRT<br>2. Inclusion of primary and recurrent tumors hampers analysis of results.<br>3. May be appropriate.	3									
	Recurrent tumors (after prior hysterectomy)	4	1	4	3	2.2	75%	Not appr	high	high	Agreed. Patients with recurrent tumors after prior hysterectomy are not eligible.
	Comments:<br>1. Inclusion of primary and recurrent tumors hampers analysis of results.<br>2. Should be included	3									

Clinical Trial Criteria	Vote: Nr of Votes	Min Rank	Max Rank	Range of ranks	Mean Rank	%Agreement with Vote	Vote Category	Agreement	Expert Panel: Exp. Panel Consensus	Exp. Panel Conclusions
**6 EXCLUSION Criteria: These conditions should be EXCLUDED from an SFRT trial** – continued										
Neoadjuvant chemotherapy	4	1	9	8	3.5	75%	Not appr	moderate	high	Agreed. Patients who have received neoadjuvant chemotherapy are not eligible.
Comment:<br>Should be concurrent	1									
Inflammatory bowel disease	4	3	8	5	5.0	50%	May be appr	low	high	Not agreed. Patients with inflammatory bowel disease are not eligible.
Comments:<br>1. At the discretion of the oncologist.<br>2. Appropriate to be excluded.	2									
Scleroderma (Systemic sclerosis)	4	3	7	4	5.0	50%	May be appr	low	high	Not agreed. Patients with scleroderma (systemic sclerosis) are not eligible.
Comments:<br>1. At the discretion of the oncologist<br>2. Consider exclusion.	3									

	Vote								Expert Panel	
**Clinical Trial Criteria**	**Nr of Votes**	**Min Rank**	**Max Rank**	**Range of ranks**	**Mean Rank**	**%Agreement with Vote**	**Vote Category**	**Agreement**	**Exp. Panel Consensus**	**Exp. Panel Conclusions**

7	**Pre-treatment Evaluations: These investigations should be required**										
	CT Abdomen/pelvis	4	4	9	5	7.0	50%	Appr<br>May be appr	low	high	Panel reconciled. Panel considers CT Abdomen/pelvis as the minimum standard. MRI for better delineation of the primary tumor, and PET/CT for evaluation for lymph node involvement and distant metastases is preferred where available.
	Comment:<br>Pre-treatment MRI and PET-CT.	1									
	MRI Abdomen/ pelvis	4	8	9	1	8.5	100%	Appr	high		
	CT Chest	4	4	7	3	6.0	50%	Appr<br>May be appr	low		
	PET/CT	4	8	9	1	8.8	100%	Appr	high		
	Comment:<br>For post treatment response.	1									

	Clinical Trial Criteria	Vote								Expert Panel	
		Nr of Votes	Min Rank	Max Rank	Range of ranks	Mean Rank	%Agreement with Vote	Vote Category	Agreement	Exp. Panel Consensus	Exp. Panel Conclusions
**8**	**Radiation Therapy – SFRT: Dose**										
	24 Gy in 3 fractions	4	6	9	3	7.5	75%	Appr	high	moderate	Agreed. For the SFRT a dose/fractionation schedule of 24 Gy in 3 fractions (BED 43.2 Gy, EQD_2 36.0 Gy, $\alpha/\beta=10$) or 16.5 Gy in 1 fraction (BED 43.7 Gy, EQD_2 36.4 Gy, $\alpha/\beta=10$) to the tumor is appropriate. More clinical experience exists with 24 Gy in 3 fractions. While there may be concerns regarding the reproducibility of SFRT dose distributions across fractions, outcome data of the 3-fractions SFRT schedule, given with IGRT, show high tumor response rates (Amendola 2020).
	15-18 Gy in 1 fraction	4	4	9	5	5.8	75%	May be appr	high	moderate	

	Vote								Expert Panel	
**Clinical Trial Criteria**	**Nr of Votes**	**Min Rank**	**Max Rank**	**Range of ranks**	**Mean Rank**	**%Agreement with Vote**	**Vote Category**	**Agreement**	**Exp. Panel Consensus**	**Exp. Panel Conclusions**

9	**Radiation Therapy – SFRT: Target volume**										
	GTV (cervical tumor) plus 2-3 mm margin	4	5	9	4	7.0	75%	Appr	high	high	Agreed. In addition, an ITV should be established because of the high mobility of the cervical target. The PTV is the ITV plus a 2-3 mm margin excluding sensitive OARs (e.g. bowel, bladder). For planning, no vertices are placed into the margin or within 2-3 mm from the GTV margin.
	GTV (lymph node mass if applicable), no margin	4	1	9	8	5.8	50%	Appr	low	high	Agreed. In rare cases where SFRT is also given to a bulky lymph node, the target volume to the involved lymph node is the lymph node GTV plus a 2-3 mm margin, margin (because of the proximity of mobile sensitive normal tissue structurers, e.g. bowel).
	Comment: It is necessary to add a margin of about 3 mm to the lymph node GTV.	1									

	Vote								Expert Panel	
**Clinical Trial Criteria**	**Nr of Votes**	**Min Rank**	**Max Rank**	**Range of ranks**	**Mean Rank**	**%Agreement with Vote**	**Vote Category**	**Agreement**	**Exp. Panel Consensus**	**Exp. Panel Conclusions**

10	**Radiation Therapy – SFRT: Normal OAR structures**										
	Dose to peripheral GTV limited to 9 Gy in 3 fractions	4	7	9	2	7.8	100%	Appr	high	high	Agreed.
	Dose to peripheral GTV limited to 6 Gy in 1 fraction	4	4	5	1	4.2	100%	May be appr	high	high	Agreed.
	Other – OAR Structures	1									
	Comment: Another possibility could be 24 Gy in 3 fractions to the vertices and the periphery as low as possible, so external beam boost after (LRT and 45-50 Gy to pelvis) is still possible.										

Clinical Trial Criteria	Vote								Expert Panel	
	Nr of Votes	Min Rank	Max Rank	Range of ranks	Mean Rank	%Agreement with Vote	Vote Category	Agreement	Exp. Panel Consensus	Exp. Panel Conclusions

11	Radiation Therapy – SFRT: SFRT technique										
	Lattice	4	5	9	4	7.8	75%	Appr	high	high	Agreed.
	GRID (collimator-based)	4	2	5	3	3.8	75%	May be appr	high	high	Agreed.
	Comment: Cit is challenging to achieve sparing of OAR for pelvic tumors.	1									
	GRID (MLC-based)	4	2	5	3	3.8	75%	May be appr	high	high	Not agreed. In the absence of any studies using GRID therapy for the definitive treatment of cervical cancer, GRID therapy is not currently recommended for a clinical SFRT trial in cervical cancer. Lattice radiation therapy (LRT) should be used.
	GRID (either within same trial)	4	1	5	4	3.5	75%	May be appr	high	high	
	Comment: The technology for cervix cancer should be only Lattice.	1									

	Vote								Expert Panel	
**Clinical Trial Criteria**	**Nr of Votes**	**Min Rank**	**Max Rank**	**Range of ranks**	**Mean Rank**	**%Agreement with Vote**	**Vote Category**	**Agreement**	**Exp. Panel Consensus**	**Exp. Panel Conclusions**

12	**Radiation Therapy – Conventional (Conv.) ERT: Dose and technique**										
	45-50 Gy at 1.8 - 2 Gy per fraction	4	7	9	2	8.2	100%	Appr	high	high	Agreed.
	IMRT	4	8	9	1	8.5	100%	Appr	high	high	Agreed.
	3D Conformal	4	2	7	5	4.2	50%	Not appr	low	high	Agreed. IMRT is preferable.
	Brachytherapy boost to a total (conv. ERT and brachytherapy) EQD2 of 80-85 Gy	4	5	7	2	5.8	75%	May be appr	high	high	Agreed. Brachytherapy with standard-of-care regimens and standard-of-care target and normal tissues doses should be given.
	Comment: Depending on the tumor response and anatomy.	1									
	Conv. ERT boost to a total of 60-66 Gy if brachytherapy not feasible	4	5	7	2	5.8	75%	May be appr	high	high	Agreed.
	Comment: Depending on OAR dose.	1									
	Conv. ERT boost to involved lymph nodes per physician discretion	4	3	9	6	6.0	50%	Appr	low	high	Agreed. Lymph node boost to involved lymph node(s), either sequential or simultaneously integrated, should be used as clinically indicated and under consideration of standard OAR constraints (see section 13).

	Clinical Trial Criteria	Vote								Expert Panel	
		Nr of Votes	Min Rank	Max Rank	Range of ranks	Mean Rank	%Agreement with Vote	Vote Category	Agreement	Exp. Panel Consensus	Exp. Panel Conclusions
13	**Radiation Therapy – Conventional ERT: OAR constraints**										
	Conventional constraints without consideration for SFRT dose	4	3	9	6	6.2	50%	Appr	low	high	Agreed. The contribution of the SFRT dose (converted to EQD2) should be included in the OAR dose.
	Comment: As long as periph-eral dose from SFRT is limited.	1									
	No more than 30% of the bowel receives >40 Gy	4	5	9	4	6.5	50%	Appr<br>May be appr	low	high	Agreed. These standard OAR constraints (with inclusion of the SFRT dose) are acceptable.
	No more than 60% of the rectum receives >40 Gy	4	5	9	4	6.5	50%	Appr<br>May be appr	low	high	
	No more than 35% of the bladder receives >45 Gy	4	5	8	3	6.2	50%	Appr<br>May be appr	low	high	
	Other Conventional ERT - OAR constraints	1								N/A	
	Comment: Consider maximum dose points.										

	Vote								Expert Panel	
**Clinical Trial Criteria**	**Nr of Votes**	**Min Rank**	**Max Rank**	**Range of ranks**	**Mean Rank**	**%Agreement with Vote**	**Vote Category**	**Agreement**	**Exp. Panel Consensus**	**Exp. Panel Conclusions**

14	**On-therapy Evaluations: Evaluate feasibility of correlative studies**										
	Toxicity assessment (weekly)	4	7	9	2	8.5	100%	Appr	high	high	Agreed.
	Correlative studies (blood, urine): pre-RT, once during RT, post-RT	4	2	9	7	6.0	50%	Appr	low	high	Agreed. Preferably within a approximately 1 day before the SFRT fraction, and within approximately 1 day after or before any cERT, and at 7-14 days.
	Correlative studies (blood, urine): pre-RT, 3 times during RT, post-RT	4	4	6	2	4.8	100%	May be appr	high	high	Agreed. The "liquid biopsy" concept is clinically feasible prospectively timed along with the treatment course.
	Comment: Depends on the study	1									
	QOL assessment	4	8	9	1	8.5	100%	Appr	high	high	Agreed.

	Vote								Expert Panel	
**Clinical Trial Criteria**	**Nr of Votes**	**Min Rank**	**Max Rank**	**Range of ranks**	**Mean Rank**	**%Agreement with Vote**	**Vote Category**	**Agreement**	**Exp. Panel Consensus**	**Exp. Panel Conclusions**

14	**On-therapy Evaluations: Evaluate feasibility of correlative studies** – continued										
	Tumor biopsy once during radiation therapy	4	1	6	5	3.2	50%	Not appr May be appr	low	high	Panel reconciled. Repeat (cervical) tumor biopsies at the time of brachytherapy or as a clinic procedure may be feasible in selected centers. However, tumor biopsies more than once during therapy are clinically challenging.
	Tumor biopsy twice during radiation therapy	3	1	3	2	2.0	100%	Not appr	high	moderate	
	Comment: For discussion	1									
	Normal tissue biopsy once during radiation therapy	3	1	4	3	2.3	67%	Not appr	high	high	Agreed. Repeat (cervical) tumor biopsies at the time of brachytherapy or as a clinic procedure may be feasible.
	Comment: For discussion	1									
	Normal tissue biopsy twice during radiation therapy	3	1	3	2	2.0	100%	Not appr	high	high	Agreed. Tumor biopsies more than once during therapy, while possible, are clinically challenging.
	Comment: For discussion	1									

	Vote								Expert Panel	
**Clinical Trial Criteria**	**Nr of Votes**	**Min Rank**	**Max Rank**	**Range of ranks**	**Mean Rank**	**%Agreement with Vote**	**Vote Category**	**Agreement**	**Exp. Panel Consensus**	**Exp. Panel Conclusions**

15	Concurrent systemic therapy: Agents										
	Weekly Cisplatin	4	6	9	3	8.0	75%	Appr	high	high	Agreed.
	Taxanes	3	2	6	4	4.7	67%	May be appr	high	high	Agreed.
	5-FU	3	2	5	3	4.0	67%	May be appr	high	high	Agreed.

Rating scale and Vote categories: 1,2,3=not appropriate -- 4,5,6=may be appropriate -- 7,8,9=appropriate

	Vote								Expert Panel	
**Clinical Trial Criteria**	**Nr of Votes**	**Min Rank**	**Max Rank**	**Range of ranks**	**Mean Rank**	**%Agreement with Vote**	**Vote Category**	**Agreement**	**Exp. Panel Consensus**	**Exp. Panel Conclusions**

16	**Concurrent Systemic Therapy: Timing**										
	Systemic therapy DURING SFRT FRACTION permitted	4	1	9	8	5.5	50%	Appr	low	high	Agreed. Concurrent Cisplatin chemotherapy begins with the first SFRT fraction.
	Neoadjuvant systemic therapy NOT allowed	4	1	9	8	5.8	50%	Appr	low	high	Agreed. Neoadjuvant systemic therapy is not permitted.

Clinical Trial Criteria	Vote								Expert Panel	
	Nr of Votes	Min Rank	Max Rank	Range of ranks	Mean Rank	%Agreement with Vote	Vote Category	Agreement	Exp. Panel Consensus	Exp. Panel Conclusions
**17 Concurrent Systemic Therapy: Immunotherapy**										
Immunotherapy as part of trial regimen	4	1	8	7	4.2	50%	May be appr	low	high	Not agreed. The first trial of SFRT in cervical cancer should use concurrent standard chemotherapy (weekly Cisplatin). Combinations with immunotherapy shall be reserved for future trials.

Clinical Trial Criteria	Vote								Expert Panel	
	Nr of Votes	Min Rank	Max Rank	Range of ranks	Mean Rank	%Agreement with Vote	Vote Category	Agreement	Exp. Panel Consensus	Exp. Panel Conclusions
**18 Post-therapy Evaluations: Clinical**										
Clinical exam	4	9	9	0	9.0	100%	Appr	high	high	Agreed.
Toxicity assessmen t	4	9	9	0	9.0	100%	Appr	high	high	Agreed.
Every 3 months (year 1-2)	4	8	9	1	8.8	100%	Appr	high	high	Agreed.
Every 4-6 months (year 3-5)	4	8	9	1	8.8	100%	Appr	high	high	Agreed.
QOL assessment	4	8	9	1	8.8	100%	Appr	high	high	Agreed.
Patient reported outcomes	4	3	9	6	7.0	75%	Appr	moderate	high	Agreed.

Clinical Trial Criteria		Vote								Expert Panel	
		Nr of Votes	Min Rank	Max Rank	Range of ranks	Mean Rank	%Agreement with Vote	Vote Category	Agreement	Exp. Panel Consensus	Exp. Panel Conclusions
19	**Post-therapy Evaluations: Imaging**										
	PET/CT 3 months post-therapy	4	7	9	2	8.2	100%	Appr	high	high	Agreed.
	MRI 1 month post-therapy	4	6	9	3	7.5	75%	Appr	high	high	Agreed.
	Additional imaging if clinically indicated (per standard of care)	3	1	8	7	5.7	67%	Appr	moderate	high	Agreed.
	Comment: PET CT 6 months and once a year after	1								high	Agreed.

20		**Knowledge gaps in SFRT for cervical cancer**
		Dose fractionation
		Little experience
	Clinical:	Better understanding of clinical feasibility
		Volume
		Better understanding of best dose, SFRT fractionation and timing between SFRT and cERT
		Better understanding of normal tissue tolerance
		Better understanding of integration of SFRT, cERT and brachytherapy
		Only patients with advanced stages have anatomical alterations caused by the tumor that are severe enough to prevent brachytherapy
		Need for studies with larger number of patients
		Advanced technology required. IGRT required, which is not available in many centers
	Physics	Technique
		Advanced technology required. IGRT required, which is not available in many centers
	Biology:	Effect of single SFRT fraction vs. multiple hypo-fractions result on clinical outcomes

21	Demographics of Voters

I am practicing or have practiced clinical SFRT in patient care.
I am practicing or have practiced clinical SFRT in cervical cancer patients.
I have presented abstract(s) on clinical SFRT including patient outcomes.
I have presented abstract(s) on clinical SFRT including patient outcomes in cervical cancer patients.
I have published scientific article(s) on clinical SFRT including patient outcomes.
I have published scientific article(s) on clinical SFRT including patient outcomes in cervical cancer patients.

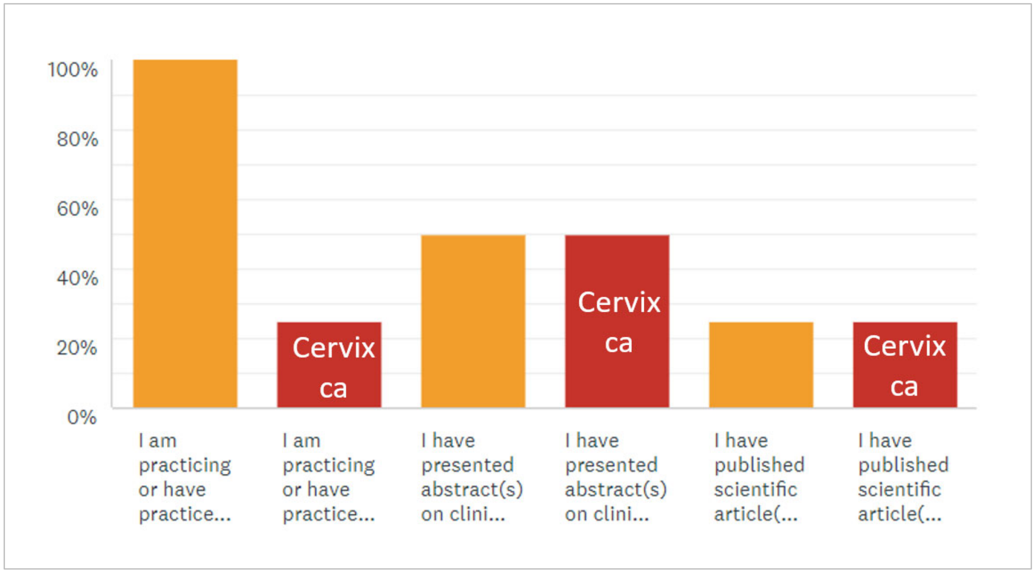

Supplement: Supplementary file 1 [file cancers-14-04267-s001.zip › cancers-14-04267-s001/Amendola_File S2_ClinTrial Cons Gyn_Consensus-Tbl (FNL).pdf]
