# Supplementary material for: An International Consensus on the Design of Prospective Clinical–Translational Trials in Spatially Fractionated Radiation Therapy for Advanced Gynecologic Cancer
Source: Cancers (Basel). 2022 Aug 31;14(17):4267. doi: 10.3390/cancers14174267 (PMC9454841; doi:10.3390/cancers14174267)
Supplement: Supplementary file 1 [file cancers-14-04267-s001.zip › cancers-14-04267-s001/cancers-1822389 File S3.pdf]

Supplementary File S3

Consensus Guideline for Clinical Trial Design:

SFRT for Primary Gynecologic Cancer

Introduction

Spatially fractionated radiation therapy (SFRT) is the treatment of tumors with highly heterogeneous dose distributions using large ablative-size dose fractions. In pilot studies, promising outcomes of unexpectedly high tumor responses with low toxicity rates have been observed with SFRT in challenging bulky, treatment-refractory recurrent/metastatic tumors [1-3]. More recent studies showed similarly favorable responses to SFRT in several malignancies treated with curative intent [4-13]. SFRT pilot data are promising in bulky advanced gynecologic cancer [10-12]. Specifically, in cervical cancer high response rates with have been seen [10 11], that mirror the results of the prior SFRT experience in other tumors [1-3].

However, no multi-institutional or prospective randomized clinical trials of SFRT have been conducted in gynecologic cancer. Such trials are important to rigorously evaluate the utility of SFRT in advanced stage, bulky gynecological cancer, which remains a therapeutic challenge with generally low tumor control and survival rates with currently available treatments.

The development of clinical trials in SFRT for gynecologic cancer are challenged by the unfamiliar dosing concepts of heterogeneous irradiation, complex and unfamiliar dosimetric and physics metrics, and the variable SFRT techniques and technologies. A consensus on a standardized approach regarding eligibility, SFRT dose prescription, dose reporting of the complex heterogeneity parameters, suitable endpoints, and the feasibility of translational trial components to elucidate the biological underpinnings of the SFRT effect, are needed to enable clinical trials in primary gynecologic cancer and facilitate broad participation and successful accrual.

The purpose of this consensus guideline effort is to develop a common approach for the design of future prospective multi-institutional clinical trials of SFRT in gynecologic malignancies.

The consensus development process is described in detail in Table 1 of the manuscript. In brief, after an initial review of the SFRT literature, a group of recognized SFRT experts rated a comprehensive set of clinical trial design categories (detailed in the guideline) through anonymous electronic voting. The draft guideline was developed by a multi-disciplinary SFRT Conesus Expert Panel for gynecologic Cancer. The voting results were shared among the Expert Panel and were iteratively reviewed and discussed to develop the draft consensus guideline for clinical trial design. The draft guideline was posted on the RSS website for public review and comment. The comments were reviewed by the Expert Panel, and after consideration of the

comments the guideline was finalized.

SFRT Clinical Trial Design Consensus Guideline for Gynecologic Cancer

The SFRT clinical trial design recommendations are informed and guided by studies of multiple disease sites that included gynecologic cancer patients [1-3], reports of individual gynecologic cancers [10 12], and by a *disease-specific* series of cervical cancer patients [11]. These published data were considered in the consensus process in conjunction with the clinician, physicist and biologist experience of the multidisciplinary Expert Panel for SFRT Clinical Trials in Gynecologic Cancer.

Eligible Disease Sites

Based on the patient characteristics of the published outcome studies [10 11], the Panel determined that cervical cancer is currently the only primary gynecologic tumor site with sufficient pilot data to warrant an SFRT clinical trial. The few reports of SFRT in other primary gynecologic malignancies consist of patients with unspecified gynecologic primary tumors that are part of larger multi-disease series containing largely palliatively treated patients [1 2]. In addition, two ovarian cancer cases have been reported, one as part of a multi-disease series [3], and one ovarian sarcoma patient in a case report [12]. More pilot clinical outcome experience would be required to justify an SFRT trial in a gynecological cancer site other than cervical cancer.

Eligibility/Exclusion criteria: Disease Stage, Tumor Size/Extent/invasion

Patients with advanced bulky cervical cancer of $FIGO_{2018}$ stages IB2–IVA with tumors of 6 cm or larger in largest diameter (by palpation and/or MR imaging) are eligible for trial enrollment (moderate consensus) based on their overall lower local control and survival outcomes. In addition, inclusion of patients with tumors of at least 5 cm in largest diameter, who are ineligible for brachytherapy because of severe anatomic distortion, such as anatomical or tumor-related severe vaginal atrophy and/or stenosis, obstructing uterine fibroid or other uterine abnormalities, and/or severe (American Society of Anesthesiologists grade IV) medical comorbidities, can be considered for enrollment (moderate consensus).

Patients with both, uninvolved and involved pelvic lymph nodes, and with involvement of either pelvic and/or para-aortic lymph nodes are eligible. Patients with involved supraclavicular lymph nodes and distant metastases are excluded (high consensus).

Eligibility/Exclusion criteria: Histology

Eligible histologies include squamous cell carcinoma, adenocarcinoma and mixed adeno-squamous carcinoma. Both HPV-positive and HPV-negative tumors are eligible. Less common biologically distinct or highly radiosensitive histologies, such as small cell neuroendocrine carcinoma, sarcoma or lymphoma are excluded (high consensus).

Eligibility/Exclusion criteria: Prior treatment

Recurrent tumors after prior surgery or prior radiation therapy. Patients with recurrent tumors after prior hysterectomy or prior definitive radiation therapy for cervical cancer are ineligible for a trial of primary cervical cancer (high consensus). Patients with bulky recurrence after hysterectomy may be considered for a separate trial of recurrent disease if no prior radiation was received. Surgical retroperitoneal, laparoscopic assisted or robotic lymph nodes dissection prior to radiation therapy is permitted.

Prior chemotherapy. Patients with prior systemic therapy (chemotherapy or targeted therapy), such as neoadjuvant chemotherapy, are not eligible because of the confounding influence of pre-trial systemic therapy on the interpretation of outcome endpoints (high consensus).

Eligibility/Exclusion criteria: Patient factors (age, toxicity risk factors)

Patients 18 years or older are eligible for enrollment, with no upper age limit as long as performance status is acceptable (generally ECOG score <2, high consensus). Patients at increased risk for normal tissue complications, such as a history of inflammatory bowel disease, or scleroderma (systemic sclerosis) should be excluded (high consensus).

Endpoints

The feasibility of delivering SFRT according to the dosimetric and physics specifications [14] (see sections Radiation Therapy: SFRT Dose), and response metrics including tumor response and local control are suitable primary endpoints. Local progression-free, metastasis-free and overall survival, toxicity and quality of life outcomes present additional clinical trial endpoints.

Stratifications

The trial cohort should be stratified according to lymph node status (uninvolved vs. involved) due to the profound prognostic impact of lymph node status in this patient population.[11]

Pre-treatment Evaluations (clinical, imaging, histologic investigations)

Pretreatment evaluation is recommended according to standard of care, including clinical examination, inclusive of pelvic exam, blood count and blood chemistries. For imaging chest/abdomen/pelvis CT is the minimum requirement. MRI to define the tumor extent in the pelvis (which can also assist in treatment planning), and PET/CT for identification of lymph node involvement and distant metastases are favored if available (high consensus).

Radiation Therapy: SFRT Dose

Based upon outcome data in cervical cancer [11] and other gynecologic malignancies [12] as well as the multi-disease studies including gynecologic cancers [1-3], two dose regimens can be considered. The most studied schedule is 24 Gy in 3 fractions on consecutive days to the tumor target in the cervix.

The dose schedule of 24 Gy in 3 fractions has been studied in 10 patients with highly advanced cervical cancer (stages IIIB-IVA with excessive tumor bulk of $\geq$7 cm and /or severe anatomical distortion) by Amendola and *al.* [11], who observed high clinical and molecular imaging response

and very low toxicity (no grade >3 short or long-term toxicity). Among the cohort, 5 patients had adjuvant hysterectomy after high tumor response, and 3 of the 5 had no residual tumor in the surgical specimen (pathologic complete response) (personal communication, Dr. B. Amendola). No brachytherapy was given in this series.

While the Panel recognizes the schedule of 24 Gy in 3 fractions as the most studied regimen, there was overall moderate consensus regarding the SFRT dose in cervical cancer. A single-fraction schedule has the potential advantage to avoid inter-fraction shifts of the high-dose peaks within the tumor, although such shifts might not have the same implication as in the conventional RT. Conversely, a 3-fraction schedule may be better tolerated by critical normal tissues. While the corresponding isoeffective single-fraction regimen may be 16.5 Gy in 1 fraction (BED 43.7 Gy, LQED 36.4 Gy, α/β=10, compared to BED 43.2 Gy, LQED 36.0 Gy, α/β=10 for 24 Gy in 3 fractions, applying radiobiologic modeling for uniform dose as an estimate), the clinical use of 16.5 Gy in 1 fraction has not been reported in cervical cancer, and the conventional BED or EUD formalisms have not been fully justified.

A single-fraction of 15 Gy Lattice radiation therapy (LRT) has been used in two cases (unpublished) as part of a recent regimen proposed by Larrea et al. [15], combined with full dose conventional radiation with concurrent chemotherapy and brachytherapy. The Panel therefore considers 15 Gy in a single fraction as a potential SFRT dose regimen in cervical cancer. However, the combination of this regimen with brachytherapy remains to be tested prospectively (see section *Radiation therapy – Conventional radiation therapy: Dose and technique: Brachytherapy*).

Standardization of the SFRT prescription dose, defined as the peak dose, is mandatory. Dosimetric and geometric characteristics of the heterogeneous dose distribution, such as dose volume histogram parameters (e.g., D10, D50, D90), vertex diameter, vertex volume and vertex distance, valley dose, and peripheral target dose must be reported according to guidelines further described in the recent LRT physics and dosimetry white paper [16].

The equivalent uniform dose (EUD) of the SFRT regimen must be determined for the trial regimen. This includes the EUD for cervical squamous cell and adenocarcinoma (using a/b = 10 Gy), and for critical normal tissues (generally a/b = 3 Gy). Current concepts favor the modified linear quadratic (MLQ) for EUD calculation because of its greater accuracy at doses of >10 Gy. However, the traditional formalism developed for whole target volume irradiation awaits justifications or modifications in the SFRT settings. Detailed models for EUD computation and qualifications/precautions are described in the recent SFRT physics guideline publications [14 16].

Radiation Therapy: SFRT Target volume

SFRT should be delivered to the primary (cervical) tumor. The GTV is the primary cervical tumor extent as defined by imaging. To account for organ motion simulation with both full and empty bladder is recommended to establish an internal target volume (ITV). For the planning target volume (PTV) a 2-3 mm margin is added to the ITV because of the proximity of mobile sensitive

normal tissue structurers, particularly small bowel, large bowel and bladder (high consensus). For Lattice therapy a Lattice volume (V_L), which is the ITV minus an inward margin that allows for the dose to drop from the dose peaks (vertices) to the periphery, should be created. Vertices should be only placed within the volume that is common for both empty and full bladder scans.

While SFRT is primarily given to the bulky primary tumor in the cervix, the delivery of SFRT to a bulky ($\geq$6 cm) lymph node or lymph node conglomerate is permitted. In the case of SFRT to a bulky lymph node, the GTV includes the lymph node mass GTV plus a 2-3 mm margin to account for the proximity of mobile sensitive normal tissue structurers (high consensus).

Radiation Therapy: SFRT: Normal Organ-at-Risk structures

For the 3-fraction regimen of 24 Gy in 3 fractions, the dose to the periphery of the PTV should be limited to no more than 9 Gy in 3 fractions (BED 18 Gy, LQED 10.8 Gy for α/β=3). For the 1-fraction regimen of 16.5 Gy the peripheral GTV dose is limited to no more 6 Gy in 1 fraction (BED 18 Gy, LQED 10.8 Gy for α/β=3) (high consensus). There is (unpublished) evidence that lower peripheral doses are achievable with Lattice therapy that maintain 2cc doses to rectum, bladder and sigmoid of < 5-6 Gy (in 3 fractions) for a prescription dose of 24 Gy in 3 fractions).

Radiation therapy – SFRT: SFRT technique

LRT should be employed as the SFRT technique. In the absence of any studies using GRID therapy for the definitive treatment of cervical cancer, GRID therapy is not currently recommended for a clinical SFRT trial in cervical cancer (high consensus).

For treatment delivery of the SFRT, it is emphasized that organ motion be managed, as further described in section *Radiation Therapy: SFRT Dose*, and daily image-guided therapy with pre-treatment cone beam CT is performed with stereotactic alignment (as customary in stereotactic body radiation therapy). CBCT imaging is particularly important due to the frequently observed rapid tumor response that may require adaptive therapy. In the series by Amendola et al. [11] adaptive therapy was needed in 7 of the 10 patients.

Radiation therapy – Conventional radiation therapy: Dose and technique

External beam radiation. SFRT is followed by conventionally fractionated radiation therapy (cERT) to a dose of 45-50 Gy at 1.8 - 2 Gy per fraction to the whole pelvis and to tumor extension beyond the pelvis (e.g., para-aortic lymph nodes), as clinically indicated (high consensus). The use of IMRT to reduce normal tissue dose is highly encouraged (high consensus) in view of the proximity of sensitive normal tissues and the overall high cumulative target doses in cervical cancer due to the use of brachytherapy (that tend to be higher than in other tumors treated with external radiation alone). In the SFRT literature for cervical cancer, the conventional doses to gross tumor PTV ranged from 39.6 to 45.0 Gy in 25 fractions Gy (combined with SFRT of 24 Gy/3 factions) [11].

Boosts to involved lymph node(s) can be delivered either sequentially (following whole pelvis

radiation) or with a simultaneously integrated boost as clinically indicated and under consideration of the OAR constraints described in section *Conventional ERT: OAR constraints*. An initial SFRT boost to a voluminous involved lymph node or matted lymph nodes is permitted as described in section *SFRT Target volume.* (high consensus). If brachytherapy is not an option, external beam either conventionally fractionated IMRT under consideration of OAR dose limits or stereotactic hypofractionated boost to the residual tumor has been used with up to 25 Gy in 5 fractions (SBRT) without complications [11].

The interval between the SFRT fractions(s) and the cERT remains an open question for SFRT in cervical cancer and other primary tumors. An interval of 1-3 days has been proposed and employed successfully in head and neck cancer and sarcoma [5 7 8]. While a 7-day interval would be more advantageous to allow immune activation for potential intra-tumoral immune or abscopal effects postulated in SFRT [17 18], this approach is hampered by the current lack of experience with longer intervals between SFRT and cERT. Furthermore, clinical experience has shown that overall protraction of the treatment course in cervical cancer is associated with a decrease in survival in standard fractionated radiation [19]. Whether this detriment in survival would apply to a lengthening of the treatment course from the addition of SFRT and the associated time interval to cERT remains unknown in cervical cancer and would require careful investigation.

Brachytherapy. There is currently minimal experience with the addition of brachytherapy to SFRT and conventional pelvic radiation/concurrent chemotherapy. The only (unpublished) experience to date has employed single-dose LRT of 15 Gy, combined with 2 fractions of intracavitary/interstitial brachytherapy (according to the EMBRACE regimen) in two highly advanced cervical cancer patients. The Panel believes that the experience with combined SFRT, cERT/concurrent chemotherapy and brachytherapy is too sparse at this time to make recommendations on the combination of brachytherapy with SFRT and cERT for a randomized trial in definitively treated cervical cancer. Brachytherapy is an indispensable component of the radiotherapeutic management of cervical cancer and ultimately will have to be incorporated into SFRT regimens for clinical trials. In the absence of solid outcome data, the concerns of added toxicity from the cumulative dose of SFRT, cERT and brachytherapy remain. The Panel therefore recommends that an initial Phase I study be conducted first, which combines SFRT with a conventional cERT regimen (with standard concurrent chemotherapy) and standard brachytherapy.

For brachytherapy, a more fractionated schedule of at least 4 fractions, as is commonly used in clinical practice, is recommended, and image guidance for the brachytherapy, preferably with MRI is strongly encouraged. For the SFRT, a stepwise dose escalation scheme from near-conventional dose (e.g. 4 Gy in 1 fraction, which has already been in used clinically to treat severe tumor hemorrhage [20] to 15 Gy, as is used in the proposed regimen [15], may be considered. A corresponding (biologically isoeffective) multi-fraction regimen, such as Amendola et al.'s [11] 3-fraction regimen, is also an option. Such a Phase I trial should contain strict guidelines for normal tissue tolerance doses, commensurate with current

recommendations from image-guided brachytherapy. Further, consideration should be given to a dosimetric feasibility study (on image-guided ERT and brachytherapy data sets) prior to a Phase I trial in clinical patients.

Radiation therapy – Conventional ERT: OAR constraints

Dose constraints to OARs for the cERT portion of treatment are recommended to follow those in standard practice, such as OAR dose limits established by the RTOG 0921 trial [21]. The contribution of the SFRT dose (converted to EQD2) is included into the determination of the OAR dose for each critical normal structure (high agreement).

On-therapy Evaluations: Evaluate feasibility

On-treatment evaluations should include standard-of-care weekly toxicity assessments, quality-of-life assessments and patient reported outcomes, and routine imaging that typically includes CBCT imaging for response assessment and adaptive therapy as needed.

Specimen collection of blood and urine before, multiple times during and after the radiation therapy course for translational correlative science studies of SFRT is feasible and should be strongly considered (high consensus). Such specimens may include investigations of immune status, such as assessments of immune cell phenotypes; circulating cytokines linked to immune activation and radiation sensitivity and circulating tumor cells, markers of senescence, and other investigations [22-25]. From a biological perspective, collection of such specimen just before SFRT, within a day after SFRT and before cERT, and 7-14 days after SFRT may be most impactful.

While repeat (cervical) tumor biopsies at the time of brachytherapy may be feasible in selected centers, tumor biopsies once or more than once during therapy, while possible, are overall clinically challenging (moderate consensus). However, collection of pre-therapy tissue is available and should be considered for translational science studies.

Systemic therapy: Agents and timing

Chemotherapy with weekly Cisplatin, the standard of care for advanced cervical cancer, should be administered concurrently with radiation therapy. For 3-fraction SFRT regimens, chemotherapy has been used concurrently with the first SFRT fraction, based on the study by Amendola et *al.* that showed minimal toxicity with a regimen of 24 Gy in 3 consecutive fractions given concurrently with Cisplatin chemotherapy. However, in other primary diseases that are treated with concurrent chemo-radiation therapy (e.g., head and neck cancer, lung cancer) concurrent chemotherapy has been started 2-3 days after SFRT, concurrently with the start of cERT. Post-radiation adjuvant (“outback”) chemotherapy, or adjuvant immunotherapy is not recommended for an initial trial.

Systemic therapy: Immunotherapy

There is no published experience with the combination SFRT and immunotherapy in cervical cancer, and the combination of immunotherapy and conventional radiation is currently considered experimental and limited to clinical trials. Therefore, an initial clinical trial of SFRT

in cervical cancer should employ standard concurrent weekly Cisplatin. Combinations with immunotherapy shall be reserved for future trials. Post-radiation adjuvant immunotherapy, which is also experimental, is not recommended for an initial trial.

Post-therapy Evaluations: Clinical, imaging

Post-therapy evaluations consist of standard-of-care physical examinations with pelvic exam for response and toxicity assessments (high consensus). Quality-of-life and patient reported outcomes are required. Recommended imaging studies include PET/CT 3 months post-therapy and MRI 1 month post-therapy. Additional imaging is performed if clinically indicated per standard of care (high consensus).

Knowledge Gaps that May be Addressed through SFRT Clinical Trials in Gynecologic Cancer

Clinical knowledge gaps centered on a better understanding of clinical feasibility, normal tissue tolerance, best dose and fractionation, and the combination of SFRT with brachytherapy, and these gaps could be improved through studies with larger patient numbers. Whether a single SFRT fraction vs. multiple fractions have differing outcomes requires investigation. A better understanding of the best time interval between SFRT and cERT may enhance strategies for engagement of the immune system and rationale for immunotherapy.

Knowledge gaps in the *physics* and *biology* aspects of SFRT are focused on SFRT technique and dose/fractionation, in particular, whether a single SFRT fraction vs. multiple hypo-fractions result in different clinical outcomes, and/or volume effects. Collection and banking of blood/urine before and after SFRT may provide insights into potential systemic reactions that currently remain a knowledge gap.

Conclusion

SFRT clinical trials in cervical cancer are feasible based on the clinical pilot experience in this disease, which continues to have poor outcomes with current therapies in advance bulky stages. However, data in other gynecologic cancers, in which radiation therapy plays a major role, are too sparse to justify a clinical trial at this time. In cervical cancer, patients with bulky tumors $\geq$6 cm squamous cell and/or adenocarcinomas, with or without lymph node involvement, or those unsuitable for brachytherapy, are eligible. LRT is favored over GRID therapy based on currently available clinical experience. While dosing in clinical pilot experience used an LRT regimen of 24 Gy in 3 fractions, a peak dose of 15 Gy in 1 LRT fraction may be acceptable; however, outcome data is sparse. SFRT is followed by conventionally fractionated external beam radiation and chemotherapy. Reporting of inhomogeneity dose parameters, particularly EUD, is highly recommended to allow robust correlation of dose parameters with clinical outcome. Standard-of-care concurrent weekly Cisplatin chemotherapy is given. Because the experience with the combination of SFRT, cERT/concurrent chemotherapy with standard-of-care brachytherapy is insufficient at this time, a Phase I trial of

LRT to test the tolerability of the combined regimen is recommended as an initial trial prior to larger-scale trials. Standard pre-, on-and post-therapy assessments and quality of life metrics should be performed. Specimen collection (blood, urine), synchronized prospectively with the treatment course, for translational correlative science studies is highly recommended. Intra-therapy tumor biopsy of the cervix may be feasible in selected centers, particularly biopsies at the time of the brachytherapy procedure.

The trial design consensus guideline presented here is based on the current status of knowledge in SFRT for gynecologic cancers. While these recommendations may provide guidance for the design of clinical trials and clinical feasibility considerations for translational studies, the field of SFRT is developing rapidly. Therefore, new data and longer-term outcome results in larger patient series may further refine, adapt or modify these initial recommendations. The clinical trial design guideline proposed here, particularly translational components, will have to be individualized by the respective investigators who develop clinical trials in SFRT.

References

1. Mohiuddin M, Fujita M, Regine WF, et al. High-dose spatially-fractionated radiation (GRID): a new paradigm in the management of advanced cancers. *Int J Radiat Oncol Biol Phys* 1999;45(3):721-7. doi: 10.1016/s0360-3016(99)00170-4
2. Mohiuddin M, Stevens JH, Reiff JE, et al. Spatially fractionated (GRID) radiation for palliative treatment of advanced cancer. *Radiation Oncocogy Investigations* 1996;4:41-47.
3. Neuner G, Mohiuddin MM, Vander Walde N, et al. High-dose spatially fractionated GRID radiation therapy (SFGRT): a comparison of treatment outcomes with Cerrobend vs. MLC SFGRT. *Int J Radiat Oncol Biol Phys* 2012;82(5):1642-9. doi: 10.1016/j.ijrobp.2011.01.065
4. Mohiuddin M, Memon M, Nobah A, et al. Locally advanced high-grade extremity soft tissue sarcoma: Response with novel approach to neoadjuvant chemoradiation using induction spatially fractionated GRID radiotherapy (SFGRT) (abstr). J Clin Oncol 2014;32:10575. *J Clin Oncol* 2014;32:10575.
5. Snider JW, Molitoris J, Shyu S, et al. Spatially Fractionated Radiotherapy (GRID) Prior to Standard Neoadjuvant Conventionally Fractionated Radiotherapy for Bulky, High-Risk Soft Tissue and Osteosarcomas: Feasibility, Safety, and Promising Pathologic Response Rates. *Radiat Res* 2020 doi: 10.1667/rade-20-00100.1 [published Online First: 2020/10/17]
6. Amendola BE, Perez NC, Wu X, et al. Safety and Efficacy of Lattice Radiotherapy in Voluminous Non-small Cell Lung Cancer. *Cureus* 2019;11(3):e4263. doi: 10.7759/cureus.4263
7. Huhn JL, Regine WF, Valentino JP, et al. Spatially fractionated GRID radiation treatment of advanced neck disease associated with head and neck cancer. *Technol Cancer Res Treat* 2006;5(6):607-12. doi: 10.1177/153303460600500608
8. Penagaricano JA, Moros EG, Ratanatharathorn V, et al. Evaluation of spatially fractionated radiotherapy (GRID) and definitive chemoradiotherapy with curative intent for locally advanced squamous cell carcinoma of the head and neck: initial response rates and toxicity. *Int J Radiat Oncol Biol Phys* 2010;76(5):1369-75. doi: 10.1016/j.ijrobp.2009.03.030
9. Choi JI, Daniels J, Cohen D, et al. Clinical Outcomes of Spatially Fractionated GRID Radiotherapy in the Treatment of Bulky Tumors of the Head and Neck. *Cureus* 2019;11(5):e4637. doi: 10.7759/cureus.4637
10. Amendola BE, Perez N, Amendola M, et al. Lattice radiotherapy with RapidArc for treatment of gynecological tumors: dosimetric and early clinical evaluations. *Cureus* 2010;2(9):1-6.
11. Amendola BE, Perez NC, Mayr NA, et al. Spatially Fractionated Radiation Therapy Using Lattice Radiation in Far-advanced Bulky Cervical Cancer: A Clinical and Molecular Imaging and Outcome Study. *Radiat Res* 2020;194(6):724-36. doi: 10.1667/RADE-20-00038.1
12. Blanco Suarez JM, Amendola BE, Perez N, et al. The Use of Lattice Radiation Therapy (LRT) in the Treatment of Bulky Tumors: A Case Report of a Large Metastatic Mixed Mullerian Ovarian Tumor. *Cureus* 2015;7(11):e389. doi: 10.7759/cureus.389

13. Amendola BE, Perez N, Amendola MA, et al. The Use of Lattice Radiation Therapy in Patients with Voluminous Tumors (abstr): Int J Radiation Oncol Biol Phys, 2020:e520.
14. Zhang H, Wu X, Zhang X, et al. Photon GRID Radiation Therapy: A Physics and Dosimetry White Paper from the Radiosurgery Society (RSS) GRID-Lattice-Microbeam-FLASH Radiotherapy Working Group. *Radiat Res* 2020 doi: 10.1667/RADE-20-00047.1
15. Larrea L, López E, González V, et al. Lattice Radiotherapy (LRT)-Spatially Fractionated Radiotherapy (SFRT): Case Report of Bulky Cervical Cancer (CC) (abstr). Radiosurgery Society Scientific Meeting 2022, .
16. Wu X, Perez N, Zheng Y, et al. The Technical and Clinical Implementation of LATTICE Radiation Therapy (LRT). *Radiat Res* 2020 doi: 10.1667/RADE-20-00066.1
17. Schoenhals JE, Seyedin SN, Tang C, et al. Preclinical Rationale and Clinical Considerations for Radiotherapy Plus Immunotherapy: Going Beyond Local Control. *Cancer J* 2016;22(2):130-7. doi: 10.1097/PPO.0000000000000181
18. Seyedin SN, Schoenhals JE, Lee DA, et al. Strategies for combining immunotherapy with radiation for anticancer therapy. *Immunotherapy* 2015;7(9):967-80. doi: 10.2217/imt.15.65 [published Online First: 20150827]
19. Perez CA, Grigsby PW, Castro-Vita H, et al. Carcinoma of the uterine cervix. I. Impact of prolongation of overall treatment time and timing of brachytherapy on outcome of radiation therapy. *Int J Radiat Oncol Biol Phys* 1995;32(5):1275-88. doi: 10.1016/0360-3016(95)00220-S
20. Kraiphibul P, Srisupundit S, Kiatgumjaikajorn S, et al. The experience in using whole pelvic irradiation in management of massive bleeding from carcinoma of the uterine cervix. *J Med Assoc Thai* 1993;76 Suppl 1:78-81.
21. Viswanathan AN, Moughan J, Miller BE, et al. NRG Oncology/RTOG 0921: A phase 2 study of postoperative intensity-modulated radiotherapy with concurrent cisplatin and bevacizumab followed by carboplatin and paclitaxel for patients with endometrial cancer. *Cancer* 2015;121(13):2156-63. doi: 10.1002/cncr.29337
22. Johnsrud AJ, Jenkins SV, Jamshidi-Parsian A, et al. Evidence for Early Stage Anti-Tumor Immunity Elicited by Spatially Fractionated Radiotherapy-Immunotherapy Combinations. *Radiat Res* 2020;194(6):688-97. doi: 10.1667/RADE-20-00065.1
23. Kanagavelu S, Gupta S, Wu X, et al. In vivo effects of lattice radiation therapy on local and distant lung cancer: potential role of immunomodulation. *Radiat Res* 2014;182(2):149-62. doi: 10.1667/RR3819.1
24. Lhuillier C, Rudqvist NP, Yamazaki T, et al. Radiotherapy-exposed CD8+ and CD4+ neoantigens enhance tumor control. *J Clin Invest* 2021;131(5) doi: 10.1172/JCI138740
25. Sathishkumar S, Dey S, Meigooni AS, et al. The impact of TNF-alpha induction on therapeutic efficacy following high dose spatially fractionated (GRID) radiation. *Technol Cancer Res Treat* 2002;1(2):141-7. doi: 10.1177/153303460200100207
